# Supplementary material for: Sub-minute prediction of brain temperature based on sleep–wake state in the mouse
Source: eLife. 2021 Mar 8;10:e62073. doi: 10.7554/eLife.62073 (PMC7939547; doi:10.7554/eLife.62073)
Supplement: Supplementary file 1. — A table showing the optimized values for each of the four parameters of Model 0 without modulation of asymptotes, and the three additional descriptive variables, as in Table 1. [file elife-62073-supp1.docx]

**Supplementary File 1:**

| **Animal** |  | **Asymptotes (**°**C)** | | |  | **Time Constants (h)** | |  | **Prior Wake-prevalence** | | |  | **Circadian** | |  | **RMS Error** (°C) | **Correlation** |
| --- | --- | --- | --- | --- | --- | --- | --- | --- | --- | --- | --- | --- | --- | --- | --- | --- | --- |
|  |  | Lower | Upper | Difference |  | Wake/REM | NREM |  | Size (h) | Shift (h) | Scale (°C) |  | Amplitude(°C) | Phase (h) |  |  |  |
| 603 |  | 33.57 | 36.19 | 2.62 |  | 0.33 | 0.21 |  | - | - | - |  | - | - |  | 0.38 | 0.89 |
| 606 |  | 33.77 | 37.11 | 3.34 |  | 0.35 | 0.26 |  | - | - | - |  | - | - |  | 0.39 | 0.89 |
| 608 |  | 31.49 | 34.20 | 2.71 |  | 0.33 | 0.23 |  | - | - | - |  | - | - |  | 0.35 | 0.88 |
| 609 |  | 33.49 | 36.68 | 3.18 |  | 0.29 | 0.16 |  | - | - | - |  | - | - |  | 0.33 | 0.95 |
| 612 |  | 31.95 | 34.90 | 2.95 |  | 0.30 | 0.17 |  | - | - | - |  | - | - |  | 0.33 | 0.94 |
| 613 |  | 31.81 | 36.78 | 4.97 |  | 0.36 | 0.81 |  | - | - | - |  | - | - |  | 0.41 | 0.85 |
| 616* |  | 33.66 | 36.51 | 2.84 |  | 0.29 | 0.17 |  | - | - | - |  | - | - |  | 0.34 | 0.94 |
| 617* |  | 33.33 | 36.77 | 3.44 |  | 0.37 | 0.43 |  | - | - | - |  | - | - |  | 0.36 | 0.90 |
| 619* |  | 34.32 | 36.70 | 2.38 |  | 0.30 | 0.12 |  | - | - | - |  | - | - |  | 0.30 | 0.94 |
| 620* |  | 35.63 | 38.76 | 3.13 |  | 0.33 | 0.31 |  | - | - | - |  | - | - |  | 0.46 | 0.86 |
| 622 |  | 34.88 | 38.53 | 3.64 |  | 0.29 | 0.28 |  | - | - | - |  | - | - |  | 0.41 | 0.91 |
| Median |  | 33.57 | 36.70 | 3.13 |  | 0.33 | 0.23 | - | - | - | - |  | - | - |  | 0.36 | 0.91 |
