# Supplementary material for: Sub-minute prediction of brain temperature based on sleep–wake state in the mouse
Source: eLife. 2021 Mar 8;10:e62073. doi: 10.7554/eLife.62073 (PMC7939547; doi:10.7554/eLife.62073)
Supplement: Supplementary file 2. — A table showing the optimized values for each of the parameters of the model after introducing a modulation of both asymptotes according to the prior wake-prevalence in the window preceding the assessment of temperature. Further details as in Table 1. [file elife-62073-supp2.docx]

**Supplementary File 2:**

| **Animal** |  | **Asymptotes (**°**C)** | | |  | **Time Constants (h)** | |  | **Prior Wake-prevalence** | | |  | **Circadian** | |  | **RMS Error** (°C) | **Correlation** |
| --- | --- | --- | --- | --- | --- | --- | --- | --- | --- | --- | --- | --- | --- | --- | --- | --- | --- |
|  |  | Lower | Upper | Difference |  | Wake/REM | NREM |  | Size (h) | Shift (h) | Scale (°C) |  | Amplitude (°C) | Phase (h) |  |  |  |
| 603 |  | 34.22 | 35.75 | 1.53 |  | 0.19 | 0.07 |  | 4.75 | -2.00 | 1.20 |  | - | - |  | 0.29 | 0.94 |
| 606 |  | 34.55 | 36.48 | 1.93 |  | 0.19 | 0.13 |  | 3.00 | -1.40 | 1.29 |  | - | - |  | 0.31 | 0.94 |
| 608 |  | 32.02 | 33.64 | 1.62 |  | 0.16 | 0.13 |  | 3.25 | -1.50 | 1.13 |  | - | - |  | 0.28 | 0.92 |
| 609 |  | 33.78 | 36.37 | 2.57 |  | 0.22 | 0.13 |  | 7.00 | -2.40 | 0.93 |  | - | - |  | 0.27 | 0.97 |
| 612 |  | 32.47 | 34.47 | 1.99 |  | 0.21 | 0.12 |  | 2.00 | -1.10 | 0.86 |  | - | - |  | 0.26 | 0.96 |
| 613 |  | 32.02 | 35.96 | 3.94 |  | 0.08 | 0.51 |  | 3.25 | -0.90 | 1.33 |  | - | - |  | 0.33 | 0.91 |
| 616* |  | 33.99 | 36.13 | 2.14 |  | 0.17 | 0.14 |  | 5.00 | -2.90 | 0.92 |  | - | - |  | 0.26 | 0.96 |
| 617* |  | 34.33 | 36.30 | 1.97 |  | 0.16 | 0.20 |  | 4.00 | -1.10 | 1.23 |  | - | - |  | 0.25 | 0.95 |
| 619* |  | 34.43 | 36.50 | 2.06 |  | 0.24 | 0.11 |  | 7.25 | -2.70 | 0.78 |  | - | - |  | 0.25 | 0.96 |
| 620* |  | 36.83 | 38.17 | 1.33 |  | 0.10 | 0.05 |  | 2.75 | -1.40 | 1.40 |  | - | - |  | 0.32 | 0.93 |
| 622 |  | 36.00 | 38.05 | 2.06 |  | 0.18 | 0.12 |  | 4.75 | -2.00 | 1.25 |  | - | - |  | 0.33 | 0.95 |
| Median |  | 34.22 | 36.30 | 1.99 |  | 0.18 | 0.13 |  | 4.00 | -1.50 | 1.20 |  | - | - |  | 0.28 | 0.95 |
