# Supplementary material for: Sub-minute prediction of brain temperature based on sleep–wake state in the mouse
Source: eLife. 2021 Mar 8;10:e62073. doi: 10.7554/eLife.62073 (PMC7939547; doi:10.7554/eLife.62073)
Supplement: Supplementary file 3. — The table shows the Pearson’s correlation coefficient (r) and root mean squared (RMS) error for five animals from the main experiment, after undergoing additional SD of shorter duration. Due to technical problems, the 2 hr SD experiment is missing for mouse number 622. See Table 1 for the individual optimized parameters used (asterisks denote KO mice). [file elife-62073-supp3.docx]

**Supplementary File 3:**

| **Animal** |  | **2-hour SD** | |  | **4-hour SD** | |
| --- | --- | --- | --- | --- | --- | --- |
|  |  | Correlation | RMS Error |  | Correlation | RMS Error |
|  |  |  |  |  |  |  |
| 616* |  | 0.96 | 0.30 |  | 0.96 | 0.33 |
| 617* |  | 0.96 | 0.32 |  | 0.95 | 0.39 |
| 619* |  | 0.96 | 0.36 |  | 0.96 | 0.38 |
| 620* |  | 0.91 | 0.36 |  | 0.93 | 0.34 |
| 622 |  | - | - |  | 0.94 | 0.40 |
